# The Leaf Extract of *Mitrephora chulabhorniana* Suppresses Migration and Invasion and Induces Human Cervical Cancer Cell Apoptosis through Caspase-Dependent Pathway

**DOI:** 10.1155/2022/2028082

**Published:** 2022-05-12

**Authors:** Wutigri Nimlamool, Sunee Chansakaow, Saranyapin Potikanond, Nitwara Wikan, Phateep Hankittichai, Jirapak Ruttanapattanakul, Phatarawat Thaklaewphan

**Affiliations:** ^1^Department of Pharmacology, Faculty of Medicine, Chiang Mai University, Chiang Mai 50200, Thailand; ^2^Department of Pharmaceutical Sciences, Faculty of Pharmacy, Chiang Mai University, Chiang Mai 50200, Thailand

## Abstract

Cervical cancer is rated to be the leading cause of cancer-related death in women worldwide. Since screening test and conventional treatments are less accessible for people in developing countries, an alternative use of medicinal plants exhibiting strong anticancer activities may be an affordable means to treat cervical cancer. *Mitrephora chulabhorniana* (MC) is the newly identified species; however, its biological functions including anticancer activities have been largely unexplored. Hence, in this study, we were interested in investigating anticancer effects of this plant on the human cervical cell line (HeLa). MC extract was profiled for phytochemicals by TLC. This plant was tested to contain alkaloids, flavonoids, and terpenes. HeLa cells were treated with MC extract to investigate the anticancer activities. Cytotoxicity and viability of cells treated with MC were determined by MTT assay and Trypan blue exclusion assay. Cell migration was tested by wound healing assay, and cell invasion was determined by Transwell assay. The level of caspase 7, caspase 9, and PARP was determined by western blot analysis. We found that the leaf extract of MC strongly reduced cancer cell survival rate. This finding was consistent with the discovery that the extract dramatically induced apoptosis of cervical cancer cells through the activation of caspase 7 and caspase 9 which consequently degraded PARP protein. Furthermore, MC extract at lower concentrations which were not cytotoxic to the cancer cells showed potent inhibitory activities against HeLa cervical cancer cell migration and invasion. *Mitrephora chulabhorniana* possesses its pharmacological properties in inhibiting cervical cancer cell migration/invasion and inducing apoptotic signaling. This accumulated information suggests that *Mitrephora chulabhorniana* may be a beneficial source of potential agents for cervical cancer treatment.

## 1. Background

In 2020, among six hundred thousand of new cases and three hundred thousand deaths caused by cancer, cervical cancer was ranked the forth as a most commonly diagnosed cancer and a leading cause of mortality in women [1]. Despite the fact that cervical cancer incidences and deaths are declining in developed countries, it remains a leading cause of cancer-related deaths in women in developing regions [2, 3]. Moreover, ICO/IARC HPV Information Center has reported that cervical cancer's morbidity and mortality are definitely on the rise. Consistent with the world trend, cervical cancer has become the second leading cancer for women in Thailand [4].

The currently used cytotoxic therapy which exerts mainly on cisplatin-based combination chemotherapeutic drugs has greatly produced beneficial response [5, 6]. However, the effective medicine options for cancer are exceptionally limited since the treatment is usually less effortable for the population, especially for developing countries. Therefore, the investigation is aimed at discovering novel active anticancer compounds from natural sources that can effectively cure the disease with reasonable cost is needed. Recently, nature-derived agents have emerged as alternative means for treating certain diseases or conditions. In particular, there are numerous antitumor agents derived from natural products [7]. A variety of plants in Thailand is valuable sources of biologically active compounds [8–13]. Many of them have been encouraged as potential chemotherapeutic candidates. For instance, we previously disclosed that some plants predominant in Thailand exhibited their strong activities against cervical cancer and ovarian cancer cell lines with the defined possible molecular mechanisms of action [11, 14, 15]. As a pace towards the discovery of possible anticancer compounds, we focused on studying a new species of plant (*Mitrephora chulabhorniana*) in the largest family of the magnolia which contains certain plant members that exhibit anticancer properties.

*Mitrephora chulabhorniana* (Annonaceae), known as Phrom Chulabhorn in Thai, is a remarkable new species discovered in a karst area in southern Thailand [16]. Different secondary metabolites including alkaloids, diterpenoids, dihydrobenzofuran lignans, and polyphenolic compounds have been reported to be concentrated in *Mitrephora* plants [17–24]. Phytochemicals derived from this genus exhibited a wide range of biological properties including antimicrobial [24], antifungal [25], antitumor [26], and cytotoxicity against human cancer cell lines [19, 20, 22, 27]. However, pharmacological activity of the new species, *M. chulabhorniana*, has not yet been explored, especially for its anticancer properties. Therefore, we aimed to evaluate whether *M. chulabhorniana* extract exhibits moderate to potent cytotoxicity against human cervical cancer, HeLa cells. To the top of our knowledge, data from our study were the first evidence to demonstrate that the extract from *M. chulabhorniana* possesses cytotoxic activities in inducing programmed cell death via activating the caspase-dependent cell death signaling pathway. Our discovery provided biological understanding of mechanism of action and may lead to the identification of possible active compounds as candidate agents for cervical cancer treatment.

## 2. Materials and Methods

### 2.1. Reagents and Chemicals

Dulbecco's modified Eagle's medium (DMEM), fetal bovine serum (FBS), Trypan blue solution, and antibiotics were purchased from Gibco™ (USA). Phosphate-buffered saline (PBS), DMSO, 3-(4,5- dimethylthiazol-2-yl)-2,5-diphenyltetrazolium bromide (MTT), 4′, 6-diamidine-2′-phenylindole dihydrochloride (DAPI), and Matrigel® Matrix were procured from Corning (USA). Primary antibodies against caspase 7, cleaved caspase 7, caspase 9, cleaved caspase 9, PARP, cleaved PARP, and actin were supplied by Cell Signaling Technology (USA). Secondary antibodies including an anti-mouse IgG conjugated with IRDye®800CW and an anti-rabbit IgG conjugated with IRDye®680RT were obtained from LI−COR Biosciences (USA). Annexin V/PI kit was purchased from ImmunoTools (Germany).

### 2.2. Preparation of Alcoholic *Mitrephora chulabhorniana* (MC) Extract

#### 2.2.1. Plant Material

*Mitrephora chulabhorniana* was collected from Surat Thani province, Thailand. The plant material was identified by the taxonomist. The voucher specimen was deposited in the Faculty of Science, Chiang Mai University (number 162). The leaves of *MC* were collected and dried in the hot air oven until the moisture was less than 10%. Then, the dried leaves were pulverized. The coarse powder of the samples was separately extracted by maceration. Specifically, 200 g of dried powder of MC leaves was extracted using 2 liters of methanol as a solvent. Maceration was performed three times, and the extract mixture was filtered through Whatman No. 1 filter paper (Sigma-Aldrich, St. Louis, MO). The filtrate was concentrated using a rotary evaporator (Eyela N-1000, Japan) to obtain a brownish syrupy mass.

### 2.3. Phytochemical Screening and Chemical Profile by Thin-Layer Chromatography

Investigation of the chemical components of the MC extract was performed by thin-layer chromatography (TLC). Chloroform : ethyl acetate : methanol (5 : 3 : 2) was used as the mobile phase. Each sample was applied to a normal phase silica gel GF254 (Merck®). After development in the chamber, the TLC plates were dried with a hairdryer. The components were detected under 254 nm and 365 nm ultraviolet (UV) light. The developed plate was sprayed with respective spray reagents (anisaldehyde, DPPH spraying reagent) and dried at 100°C in a hot air oven. Rf values were used to compare the distances of the unknown spots and calculated as follows: Rf = migration distance of spot/migration distance of solvent. The extract was subjected to certain analysis phytochemical tests to determine the major chemical nature of the extract. In particular, different assays were conducted to detect the existence of alkaloids, tannins, hydrolysable tannins, condensed tannins, glycosides (including flavonoid), terpenoids, and proteins.

### 2.4. Cell Lines and Determination of Cytotoxicity by Cell Viability Assay

Human HeLa cell line (HeLa 229 (ATCC®CCL-2.1TM)) and mouse 3T3-L1 fibroblast (CL-173) used in this study were obtained from the American Type Culture Collection (ATCC) (USA). These two types of cells were cultivated in DMEM containing 10% (*v*/*v*) FBS and antibiotics (100 U/mL penicillin and 100 *μ*g/mL streptomycin) in a 37°C humidified incubator with 5% (*v*/*v*) CO_2_. Cell viability and the optimal dose (the half maximal inhibitory concentration, IC50) of MC extract for HeLa and 3T3-L1 cells were determined using MTT assay as described previously [15, 28]. Specifically, the cells were grown for 24 h in complete media before treated with MC extract. The cells were seeded into 96-well plates at a density of 1 × 10^4^ cells/well in 0.2 mL of complete media. After 24 h of incubation, the cells were exposed with various concentration of MC extract (0-1000 *μ*g/mL) for 48 h. Treatment with DMSO (0-0.5%) was included as vehicle control. At the end of the treatment, 25 *μ*L of MTT solution (5 mg/mL) was added to each well, and the plate was incubated for 1 h at 37°C. The absorbance was measured at 450 nm wavelength using a microplate absorbance reader (BioTek Instruments, USA) relative to that of untreated control in triplicate experiments.

### 2.5. Trypan Blue Exclusion Assay

On the basis that viable cells contain strong membrane integrity whereas dead cells lost their membrane permeability, we used Trypan blue to stain dead cells since this dye can freely diffuse into them. Cells at a density of 0.05 × 10^6^ cells/well were seeded in 24-well plates and incubated with MC extract at its IC_50_ concentrations (125 *μ*g/mL) or DMSO at 0.0625% as a vehicle control. Cells were harvested after 0, 6, 24, and 48 h of incubation. Trypan blue solution was added to the cell suspensions in a ratio of 1 : 1. Total cells and dead cells (stained in blue) were counted using haemacytometer. The percentage of living cells and dead cells was calculated.

### 2.6. Wound Healing Assay for Examining Cell Migration

Inhibition of migration activity in HeLa cells treated with MC extract was measured by wound-healing assay. The cells were seeded in 24-well plates in complete media until it reached 95-100% confluent. A 200 *μ*L pipette tip was used to scrape the cell monolayer in a vertical and horizontal cross-line to create a “scratch” to each well. The center of the cross, where the 2 scratch lines meet, was used to position the center of the wound gap. The wells were washed once with growth medium to clear any detached cells and then refilled with the media (untreated (UT)) or treatment media (media containing MC extract at 15, 30, and 60 *μ*g/mL or DMSO at 0.03125%). Cell migration (wound closure) was examined using a phase-contrast microscope at 0 h (immediately after scratch), 24 h, and 48 h. Wound closure was measured, and cell migration was quantified. Each treatment was done in triplicate, and each experiment was repeated at least three times.

### 2.7. Cell Invasion Assay

The effects of MC extract on HeLa cell invasion were explored by Transwell invasion assay using Cell Culture Inserts (SPL Life Sciences, Korea). The polycarbonate invasion chambers (8 *μ*m pore size) were coated with Matrigel® Matrix per well and incubated at room temperature (RT) for 1–4 h. Then, HeLa cells at a density of 0.25 × 10^6^ cells per well were seeded on Matrigel in serum-free media alone (control) or serum-free media with the presence of 15, 30, or 60 *μ*g/mL of MC extract. The invasion chambers were put into the wells (the lower chambers) containing DMEM with 10% FBS and incubated for 24 h. Cells were then fixed with absolute methanol for 5 min at RT and stained with 0.5% crystal violet for 15 min. After three washes with water, cells in the invasion chambers were removed with cotton swab, and the images of the stained cells attached at the other site of the invasion chamber were taken and analyzed with the ImageJ software.

### 2.8. Apoptosis Assay by Annexin V/PI Staining and Flow Cytometry

Cell apoptosis was examined using FITC-annexin V (ImmunoTools, Germany) and propidium iodide (PI) (Sigma, United States). After treatment with MC extract at different concentrations (62.5, 125, and 250 *μ*g/mL) for 24 h, cells (approximately 1 × 10^6^ cells/mL) were harvested by trypsinization and washed in PBS one time by centrifugation. The supernatants were discarded, and cells were resuspended in 1x annexin-binding buffer. Then, Annexin V and PI were added to the cell suspension, and cells were incubated at RT for 15 min. To determine the percentage of apoptotic cell death, stained cells were analyzed immediately by flow cytometry using a flow cytometer DxFLEX from Beckman Coulter (Indianapolis, IN, United States). Data analysis was performed using the CytExpert for the DxFLEX software.

### 2.9. Western Blot Analysis

HeLa cell lysates were prepared by adding 1x reducing Laemmli buffer into the sample dishes. Samples were collected and heated at 98°C for 10 min and then separated in a 10% gel by SDS–PAGE and electroblotted onto nitrocellulose. Membranes were blocked with 5% nonfat dry milk in TBS-T (0.02 mol/L Tris-HCl, pH 7.6, 0.137 mol/L NaCl, and 0.1% (*wt*/*vol*) Tween 20) at room temperature for 1 h. Membranes were then incubated with primary antibodies at 4°C overnight. After three washes with TBS-T, membranes were incubated with secondary antibodies for 2 h at RT. The western blot protein bands were illustrated by using an Odyssey® CLx Imaging System (LI−COR Biosciences, USA).

### 2.10. Statistical Analysis

Data were analyzed by one-way ANOVA followed by the Tukey-Kramer multiple comparisons test and expressed as the mean ± standard deviation (SD). *p* values less than 0.05 were considered statistically significant.

## 3. Results

### 3.1. Phytochemical Screening for the Leaves of *M. chulabhorniana* (MC)

**Figure 1:**
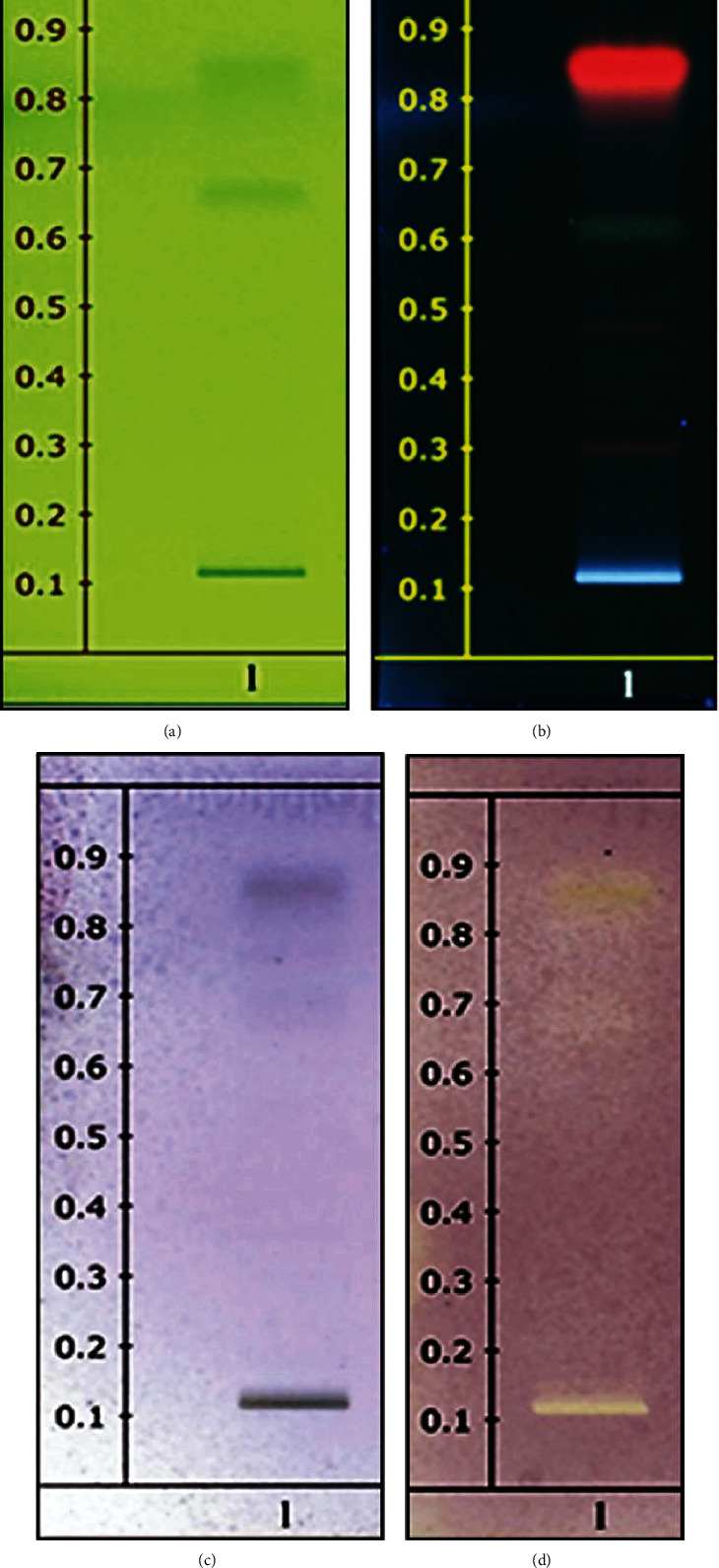
TLC chromatogram of methanolic extract of *M. chulabhorniana*. (a) Methanolic extract of leaves observed under UV at 254 nm. (b) Methanolic extract of leaves observed under UV at 365 nm. (c) Methanolic extract of leaves observed by using anisaldehyde-sulfuric acid spraying reagent. (d) Methanolic extract of leaves observed by using DPPH spraying reagent. Data are representatives of three individual replicates.

The chemical profile was performed with the suitable mobile phase and visualized under UV 254 nm and 365 nm. The TLC chromatogram of the methanolic extract was observed as shown in Figure 1. The methanolic extract of the leaves of M. chulabhorniana did not consist of apigenin, kaempferol, quercetin, and rutin compared to the standard Rf value of known flavonoid standards. In this study, TLC chromatogram did not indicate the chemical components, but the fingerprinting can be used to analyze the quantification of herbal products and identification for further study (Figure 1). However, our phytochemical test for major chemical groups revealed that the methanolic extract of the leaves of M. chulabhorniana contained alkaloids, flavonoids, and terpenes (Supplementary Table 1). On the basis that MC extract possesses anticancer properties, complete identification and characterization of certain active compounds responsible for anticancer activities of this plant should be further evaluated.

### 3.2. *Mitrephora chulabhorniana* (MC) Extract Inhibits the Growth of HeLa Cervical Cancer Cells

**Figure 2:**
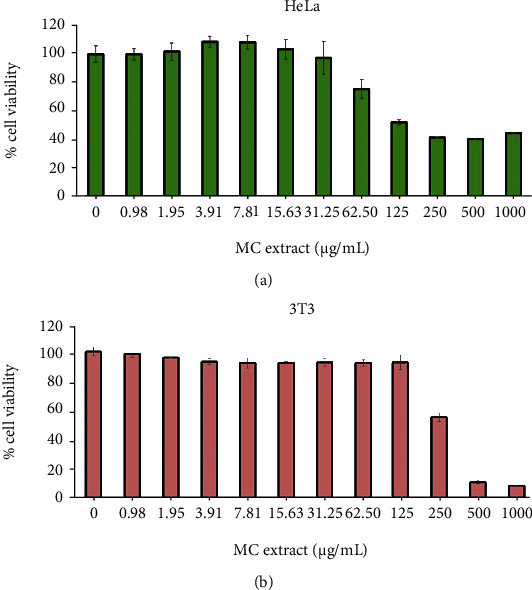
Effects of the leaf extract of MC on HeLa cervical cell and fibroblast cell viability. (a) MTT assay for testing cell viability of HeLa cells treated by MC extract at varied concentrations (0-1000 *μ*g/mL) for 48 h. (b) MTT assay for cell viability of 3T3 fibroblast cell for 48 h. Data are representatives of three individual replicates.

To evaluate the effects of the leaf extract of MC on cervical cell growth, HeLa cells were directly treated with 0, 0.98, 1.95, 3.91, 7.81, 15.63, 31.25, 62.5, 125, 250, 500, and 1000 *μ*g/mL of MC extract for 48 in complete media before subjected to MTT assay. As shown in Figure 2(a), incubation of HeLa cells with MC extract for 48 h inhibited the growth of cells in a dose-dependent manner. The concentration of MC extract that reduced HeLa cell growth to 50% was approximately 125 *μ*g/mL. Therefore, the half-maximal inhibitory concentration (IC_50_) of MC extract was 125 *μ*g/mL. MC extract at 250 *μ*g/mL reduced cell growth to be below 40%, and this cytotoxic effect of MC extract was seen to be similar to the groups treated with the extract at 500 or 1000 *μ*g/mL. We observed that DMSO had no effect on growth and viability of HeLa cells (data not shown).

When the effect of MC extract on cell viability was tested in a different cell line, 3T3, which was a fibroblast cell line, we found that MC extract at 125 *μ*g/mL had no significant effect on 3T3 cell growth (Figure 2(b)). Specifically, MC extract at 250 *μ*g/mL started to decrease 3T3 cell growth to approximately 60%. These results suggest that HeLa cervical cancer cells are more sensitive to MC treatment than a noncancerous 3T3 fibroblast cells. Additionally, MTT screening was conducted in four other cancer cell lines. Those included HN31 cell (a metastatic squamous cell carcinoma of pharynx from lymph node site), HepG2 cell (an immortal cell line derived from the liver tissue), SKOV3 cell (an ovarian cancer cell line derived from the ascites), and TOV21G (ovarian primary malignant adenocarcinoma). MTT results in these four cancer cell lines (Supplementary Figure 1) showed that in response to MC extract treatment, all four cancer cell lines exhibited a dose-dependent decrease in cell viability. Among these cancer cell lines, when we assayed percent cell viability and nuclear fragmentation, HeLa cervical cancer cells were the most sensitive to the extract at same concentrations.

### 3.3. MC Extract Induces Morphological Changes and Nuclear Fragmentation in HeLa Cervical Cancer Cells

**Figure 3:**
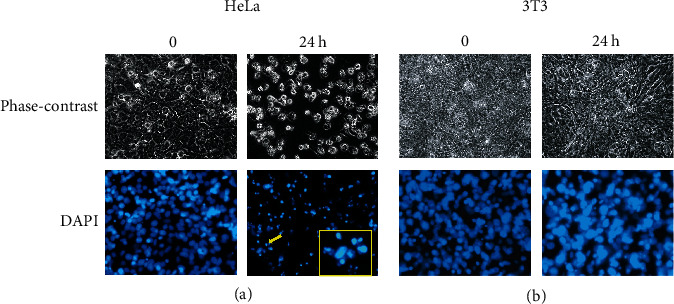
Effects of the leaf extract of MC on the cell morphological changes and nuclear fragmentation of HeLa cervical cancer cells and 3T3 fibroblast cells. (a) HeLa cells were treated with 125 *μ*g/mL of MC extract for 24 h. (b) 3T3 fibroblast cells were treated with 125 *μ*g/mL of MC extract for 24 h. Micrographs were taken at 0 and 24 h by a bright-field microscope (phase-contrast) and a fluorescent microscope to observe DNA fragmentation stained by DAPI (blue). Yellow arrow indicates DNA fragmentation, and the indicated cell is magnified in the right corner with yellow border. Data are representatives of three individual replicates.

The results from MTT assay showing that MC extract significantly inhibited HeLa cell growth over the course of 48 h led us to hypothesize that MC extract can induce HeLa cervical cell death. Therefore, we designed an experiment to observe the effects of MC on HeLa cancer cell alterations. The morphologies of the untreated and MC treated cells were compared under a phase-contrast microscope. The morphology of HeLa cells drastically changed after treatment with 125 *μ*g/mL of MC extract for 24 h (Figure 3(a)). Specifically, at the beginning of MC extract treatment (0 h), HeLa cells exhibited adherent epithelial characteristics, but after 24 h of MC extract treatment, many cells detached from the surface of the dish, and the remaining cells attaching to the surface presented the round-shape appearance and the changed pattern of light reflection under the phase-contrast microscope (Figure 3(a)). When we stained MC extract-treated HeLa cells with DAPI, we found that at the beginning of the treatment, the nuclei of the cells were intact and exhibited normal morphology (Figure 3(a)). However, the nuclei of HeLa cells presented nuclear fragmentation and a decrease in their volume after 24 h of MC extract treatment (Figure 3(a)). These morphological changes suggest that MC extract-treated HeLa cells preceded apoptosis. Similar experiments were performed in 3T3 fibroblast cells, and we did not notice any effects of MC extract on the cell morphology and the characteristics of their nuclei at both 0 h and 24 h posttreatment (Figure 3(b)). We also monitored nuclear fragmentation to confirm the effects of the extract at the selected concentration (125 *μ*g/mL) in HN31, HepG2, SKOV3, and TOV21G cells, and we found that these cell lines were less sensitive to MC extract in terms of induction of nuclear fragmentation (Supplementary Figure 2).

### 3.4. MC Extract Activates Apoptotic Cell Death in HeLa Cervical Cancer Cells

**Figure 4:**
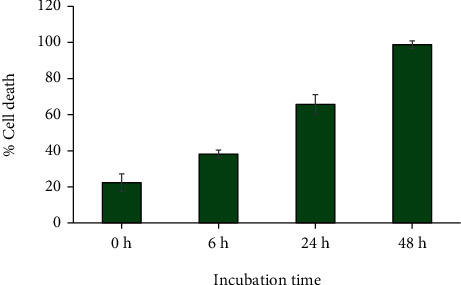
Effects of MC extract on HeLa cell death determined by Trypan blue exclusion assay. HeLa cells were treated with 125 *μ*g/mL of MC extract over the course of 48 h, and dead cells were stained by Trypan blue and quantified. Data are representatives of three individual replicates.

The effects of MC extract on changes of HeLa cell morphology and induction of nuclear fragmentation strongly suggest that MC extract may induce cell death through activating apoptosis signaling. To examine this hypothesis, we first performed Trypan blue exclusion assay to quantify the percent of dead cells after MC extract treatment. Results clearly demonstrated that MC extract at 125 *μ*g/mL could induce number of Trypan blue-positive HeLa cells over the course of 48 h in a time-dependent manner. Specifically, the percent of Trypan blue-positive HeLa cells at 0 h was around 20% which was approximately equal to that of the DMSO vehicle control group (data not shown), and the dead cells were increased to 40%, 60%, and 100% at 6 h, 24 h, and 48 h, respectively (Figure 4.).

**Figure 5:**
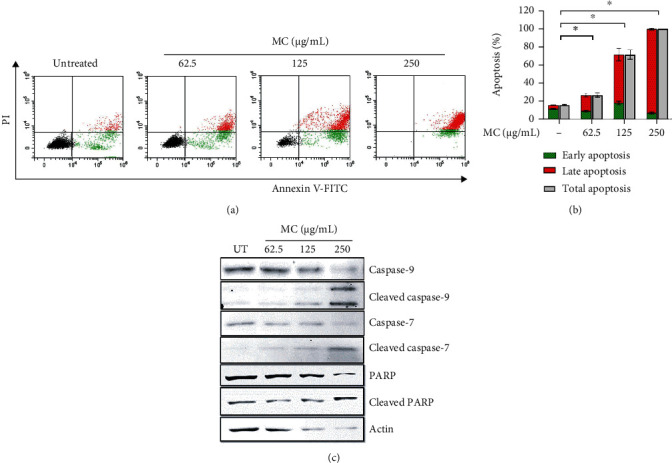
Effects of MC extract on stimulating caspase-dependent death signaling pathway. (a) HeLa cells were treated with MC extract at different concentrations (UT: untreated; 62.5, 125, and 250 *μ*g/mL) for 24 h, subjected to Annexin V/PI staining and flow cytometry. (b) Quantitative analysis of apoptotic HeLa cells from Annexin V/PI staining and flow cytometry. (c) Full-length and cleaved caspase 9, caspase 7, and PARP were detected by western blot analysis. Actin was used as an internal control. Data are representatives of three individual replicates.

To further verify the relevant signaling pathway in which MC extract sensitized HeLa cervical cancer cells to death, we determined apoptotic cell death by staining cells with Annexin V/PI and performed flow cytometry analysis. As expected, MC extract induced early and late apoptosis in HeLa cells in a concentration-dependent fashion (Figure 5(a)). Quantitative analysis demonstrated that total apoptotic cell death was induced to be approximately 20%, 70%, and 90% in cells treated with MC extract at 62.5, 125, and 250 *μ*g/mL, respectively (Figure 5(b)). Furthermore, we detected the activation of caspases and their executive substrate, PARP, by western blot analysis. As shown in Figure 5(c), MC extract could induce the cleavage of full-length caspase 9 and caspase 7. Thus, the full-length (inactive) level of these two enzymes was reduced in a concentration-dependent manner. Consistently, the active forms of caspase 9 and caspase 7 were increased when the concentration of MC extract was increased. Generally, when the executive caspase such as caspase 7 is activated, the enzyme can further degrade several different downstream substrates including PARP protein. As expected, PARP was degraded in HeLa cells treated with MC extract, and the degradation was higher when the concentration of the extract was increased (Figure 5(c)). These data confirm that MC extract induces apoptotic death of HeLa cervical cancer cells through caspase-dependent pathway. Western blot data from three independent experiments are shown in supplementary figure 3.

### 3.5. MC Extract Inhibits HeLa Cervical Cancer Cell Migration

**Figure 6:**
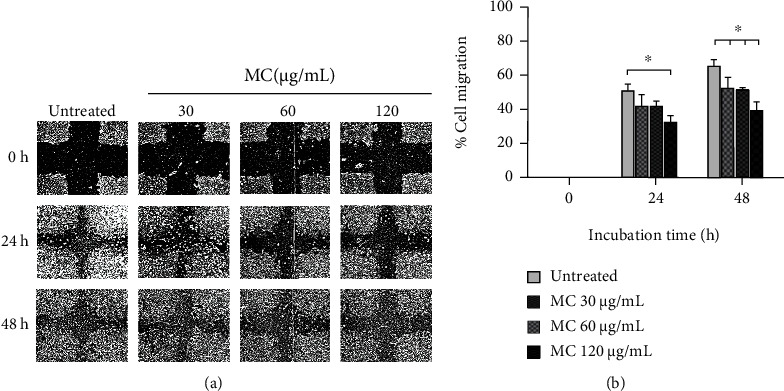
Effects of MC on HeLa cell migration. (a) Scratch wound healing assay of HeLa cells treated with MC extract at 15, 30, and 60 *μ*g/mL over 48 h. (b) Quantitative analysis of the percent HeLa cell migration after treated with MC extract at 15, 30, and 60 *μ*g/mL over 48 h. ^∗^*p* < 0.05 in comparison to the untreated group. Data are representatives of three individual replicates.

Beside the effects of MC extract on inducing apoptosis in human cervical cells, we also evaluated its effects (at low concentrations) on cervical cancer cell migration. The results from scratch wound healing assay showed that MC extract at 60 *μ*g/mL could significantly inhibit HeLa cell migration (approximately 40% inhibition) at 24 h and 48 h (Figure 6). Although the migration rate of the groups treated with MC extract at 15 and 30 *μ*g/mL was not significant, the results exhibited the reduction trend of cell migration over the course of 24 and 48 h (Figure 6).

### 3.6. MC Extract Suppresses HeLa Cervical Cancer Cell Invasion

**Figure 7:**
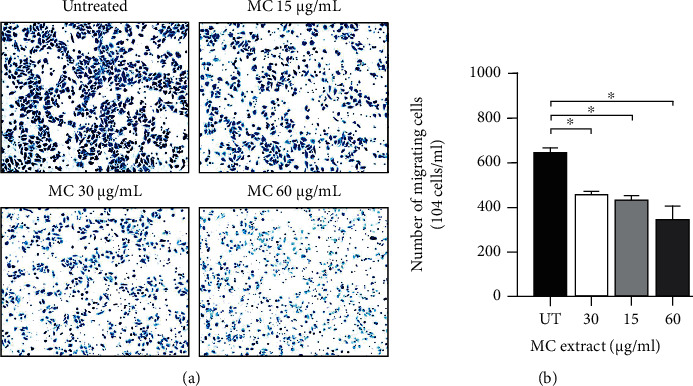
Effects of MC extract on HeLa cell invasion. (a) HeLa cells were treated with MC extract at 15, 30, and 60 *μ*g/mL for 24 h and tested for cell invasion by using Transwell invasion assay. (b) Quantitative analysis for the number of the invading HeLa cells after 24 h of MC extract treatment. ^∗^*p* < 0.05 in comparison to the untreated (UT) group. Data are representatives of three individual replicates.

Since we observed that MC could inhibit HeLa cervical cancer cell migration, we further hypothesized that the extract may also inhibit cancer cell invasion. Therefore, we performed Transwell invasion assay. Data from this experiment clearly showed that MC extract significantly suppressed numbers of invading cells in a dose-dependent manner. Specifically, about 6.0 × 10^6^ cells/mL of the untreated cells could migrate to the lower chamber, but MC extract at 15, 30, and 60 *μ*g/mL could reduce the number of HeLa cell invasion to approximately 4.5 × 10^6^ cells/mL (25% reduction), 4.0 × 10^6^ cells/mL (30% reduction), and 3.0 × 10^6^ cells/mL (50% reduction), respectively (Figure 7). These data indicate that MC extract has a potential to suppress cervical cancer cell invasion.

## 4. Discussion

For cervical cancer, the best way to reduce the burden and the associated death rate of this disease is to screen for HPV lesions through HPV testing and Pap smears and prevent by vaccination [29]. Although screening program and vaccination have been promoted for preventing the incidence of cervical cancer, this type of cancer is still the leading cause of cancer-related death in young women [30, 31]. This may be due to the fact that routine screening may not be widely available in many underdeveloped countries [32], and vaccination is currently limited to young people [33]. Conventional therapies including radiation, chemotherapy, and surgery are major means for patients; however, most of these conventional treatment can cause adverse effects (ranging from mild to severe) to the patients [34]. Medicinal plants have been extensively studied with the ultimate goal of discovering and developing novel anticervical cancer agents. For instance, the ethanolic extract of *Kaempferia parviflora* which contained certain kinds of methoxyflavones was reported to exhibit strong effects on the growth and survival of cervical cancer cells [14, 15] and ovarian cancer cells [11]. Numerous plants and their active compounds have been approved to be effective for many cancers since they can interfere with cancer homeostasis [35].

One genus that has been reported to show inhibitory effects on cancers is *Mitrephora* [19, 20, 22, 26, 27]. However, anticancer investigation for a new species, *Mitrephora chulabhorniana* (MC), has not been reported. Considering the cytotoxic effects of this genus, we believe that *Mitrephora chulabhorniana* may possess conserved pharmacological activities against cancer cells. Therefore, we focused on exploring the anticancer effects of *Mitrephora chulabhorniana* on cervical cancer cells using HeLa cervical cell line as a model for studying. Data from our study showed that in comparison to a noncancerous cell line, 3T3 fibroblast, MC extract significantly reduced cell viability of HeLa cervical cancer cells with the IC50 being approximately 2-fold lower than that for 3T3 fibroblast cells and four other cancer cell lines, including HN31, HepG2, SKOV3, and TOV21G cells. The reduction in metabolic activity of MC extract-treated HeLa cells assayed by MTT pointed to the possibility that MC extract may cause reduced cell viability through inducing cell death. Our observation by phase contrast microscopy confirmed that MC extract dramatically caused HeLa cell morphological changes over the course of 24 h. Specifically, the morphology of epithelial cells adhering to the culture dish surface changed to the round-shape with different pattern of light reflection, and significant number of cells detached from the surface. These data suggest that MC extract interferes with the normal physiological conditions of HeLa cervical cancer cells. However, the extract at the same concentration did not cause any change in the morphology of 3T3 fibroblast cells over the same treatment duration, confirming that these cells are less sensitive to MC extract in comparison to HeLa cervical cancer cells. We further investigated the morphological changes at the nuclear level by staining the nuclei of the cells with DAPI which is a fluorescent dye used to probe DNA. As expected, HeLa cells treated with MC extract for 24 h exhibited dramatic reduction in the nucleus size, compared to their original volume at 0 h of treatment. Importantly, MC extract strongly induced irregular nuclear structure pattern where it was clearly shown at the higher magnification to be nuclear fragmentation. Again, 3T3 fibroblast cells were irresponsive to MC extract as seen by their intact nuclei. These results verified that HeLa cervical cancer cells but not 3T3 noncancerous cells are susceptible for cellular stresses in response to MC extract. Nuclear fragmentation is a distinctive characteristic of dyeing cells, especially the ones undergoing apoptotic cell death [36]. As we anticipated, when MC extract-treated HeLa cells were stained with Trypan blue and counted for the proportion of dead cells, we found that MC extract significantly induced percent cell death in a time-dependent fashion over the course of 48 h. Further investigation at the molecular level indicated that MC extract induced HeLa cervical cancer cell death through activation of caspase-dependent pathway which is a major route of apoptotic cell death [37, 38]. It is possible that the extract may function to decrease the activation of growth and survival signal transduction pathways including MAPK [39] and PI3K/AKT [40] signal transduction pathways in response to growth factors or inflammatory cytokines, and these pathways are required for cancer cell proliferation and survival. Future identification of relevant signal transduction pathways by using molecular techniques as well as rational redesign of a functional protein kinase-substrate interaction [41] is required to accurately probe the cellular function which would explain how MC extract interferes with cervical cancer cell signaling.

Cellular responses upon treatment with MC extract at concentrations close to its IC_50_ could cause extensive cervical cancer cell death. This event directed us to hypothesize that MC extract at lower concentrations may have inhibitory effects on HeLa cervical cancer cells in other aspects associated with the cancer cell behavior. As it is well-known that migration and invasion are crucial steps of tumor progression [42, 43], we determined whether MC extract can suppress HeLa cell migration and invasion. Scratch wound healing assay and Transwell migration assay evidently demonstrated that MC extract at nontoxic ranges of concentration could effectively inhibit HeLa cervical cancer cell migration and invasion in a dose-dependent pattern. These findings suggest that MC extract may help suppress cervical cancer metastasis.

## 5. Conclusions

**Figure 8:**
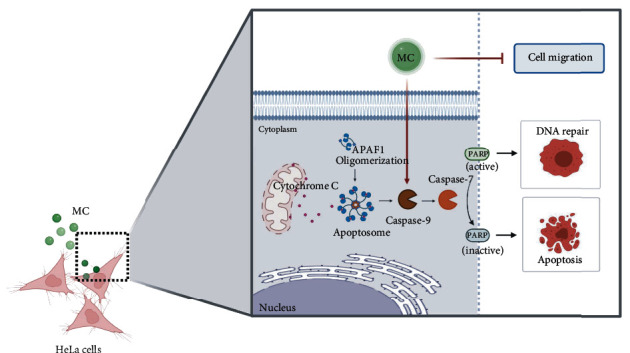
Explanatory image proposing that MC extract initiates cell apoptosis signaling and suppress migration/invasion of human cervical cancer cells, HeLa. The graphic was created with http://biorender.com/.

Taken together, like other members of plants in the same genus, our current study provided accumulated evidence that *Mitrephora chulabhorniana* possesses anticancer properties by suppressing cervical cancer cell migration and invasion as well as inducing apoptosis (as shown in schematic Figure 8). This plant may be a good candidate for isolating critical compounds in which we believed to be conserved among the plant's genus. Those potential compounds may include the terpenoids, alkaloids, and flavonoids. Further investigation of this plant and its active compounds for all pharmacology aspects related to cervical cancer cell biology would contribute greatly to the development of alternative anticervical cancer therapeutic agents.

## Data Availability

The data presented in this study are available in this article.

## Abbreviations

MC: *Mitrephora chulabhorniana*
TLC: Thin-layer chromatography
MTT: 3-(4,5-Dimethylthiazol-2-yl)-2,5-diphenyltetrazolium bromide
IC_50_: Half maximal inhibitory concentration value
DMSO: Dimethyl sulfoxide
PARP: Poly (ADP-ribose) polymerase.

## Supplementary Materials

Supplementary Materials Supplementary data associated with this article can be found in the Supporting Information. (*Supplementary Materials*). Supplementary Figure 1: effects of the leaf extract of MC on the cell viability of (A) HN3, a metastatic squamous cell carcinoma of pharynx from lymph node site; (B) HepG2 cell, an immortal cell line derived from the liver tissue; (C) SKOV3, an ovarian cancer cell line derived from the ascites; and (D) TOV21G, ovarian primary malignant adenocarcinoma. MTT assay of four cancer cell lines treated by MC extract at varied concentrations (0-1 mg/mL) for 48 h. Data are representatives of three individual replicates. Supplementary Figure 2: effects of the leaf extract of MC on the cell morphological changes and nuclear fragmentation of (A) HN31 cells, (B) HepG2 cells, (C) SKOV3 cells, and (D) TOV21G. Cancer cells were treated with 125 *μ*g/mL of MC extract for 24 h. Micrographs were taken at 0 and 24 h by a bright-field microscope (phase-contrast) and a fluorescent microscope to observe DNA fragmentation stained by DAPI (blue). Data are representatives of three individual replicates. Supplementary Figure 3: three individual replicates of western blot analysis of HeLa cells treated with different concentrations of MC extract showing caspase 9, cleaved (activated) caspase 9, caspase 7, cleaved (activated) caspase 7, PARP, cleaved PARP, and actin. DMSO was used as a vehicle control. M = protein markers. Supplementary Table 1: phytochemical tests were evaluated for major chemical groups in MC extract, e.g., flavonoids, alkaloids, glycosides, proteins, and terpenoids using chemical reagents. (+): positive result; (-): negative result.
