# Supplementary material for: The Leaf Extract of Mitrephora chulabhorniana Suppresses Migration and Invasion and Induces Human Cervical Cancer Cell Apoptosis through Caspase-Dependent Pathway
Source: Biomed Res Int. 2022 May 12;2022:2028082. doi: 10.1155/2022/2028082 (PMC9152413; doi:10.1155/2022/2028082)
Supplement: Supplementary Materials — Supplementary data associated with this article can be found in the Supporting Information. (Supplementary Materials). Supplementary Figure 1: effects of the leaf extract of MC on the cell viability of (A) HN3, a metastatic squamous cell carcinoma of pharynx from lymph node site; (B) HepG2 cell, an immortal cell line derived from the liver tissue; (C) SKOV3, an ovarian cancer cell line derived from the ascites; and (D) TOV21G, ovarian primary malignant adenocarcinoma. MTT assay of four cancer cell lines treated by MC extract at varied concentrations (0-1 mg/mL) for 48 h. Data are representatives of three individual replicates. Supplementary Figure 2: effects of the leaf extract of MC on the cell morphological changes and nuclear fragmentation of (A) HN31 cells, (B) HepG2 cells, (C) SKOV3 cells, and (D) TOV21G. Cancer cells were treated with 125 μg/mL of MC extract for 24 h. Micrographs were taken at 0 and 24 h by a bright-field microscope (phase-contrast) and a fluorescent microscope to observe DNA fragmentation stained by DAPI (blue). Data are representatives of three individual replicates. Supplementary Figure 3: three individual replicates of western blot analysis of HeLa cells treated with different concentrations of MC extract showing caspase 9, cleaved (activated) caspase 9, caspase 7, cleaved (activated) caspase 7, PARP, cleaved PARP, and actin. DMSO was used as a vehicle control. M = protein markers. Supplementary Table 1: phytochemical tests were evaluated for major chemical groups in MC extract, e.g., flavonoids, alkaloids, glycosides, proteins, and terpenoids using chemical reagents. (+): positive result; (-): negative result. [file 2028082.f1.docx]

**Supplementary data**

**Manuscript ID 2028082**

The Leaf Extract of *Mitrephora chulabhorniana* Suppresses Migration and Invasion and Induces Human Cervical Cancer Cell Apoptosis Through Caspase-Dependent Pathway


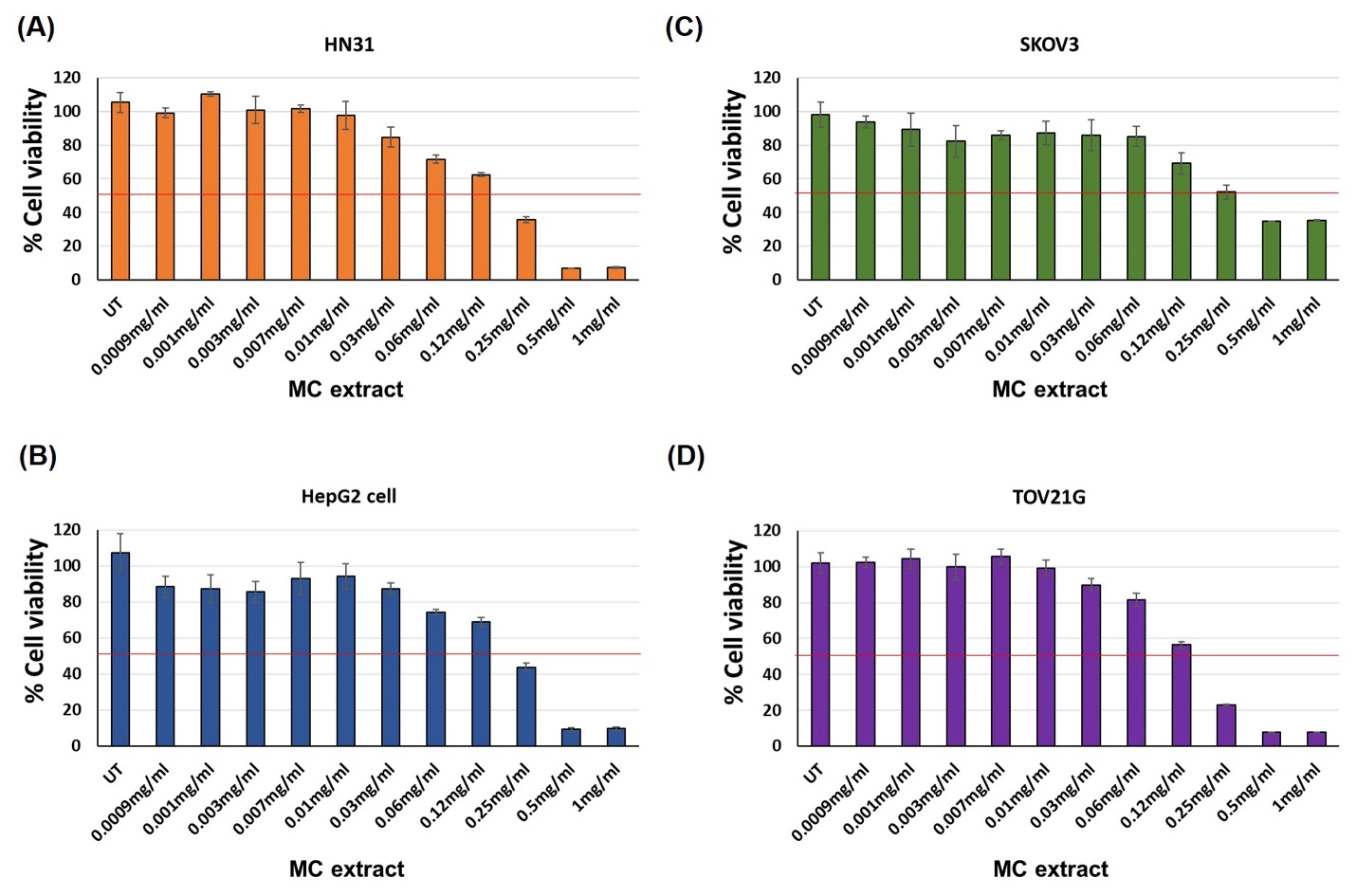


**Supplementary Fig 1.** Effects of the leaf extract of MC on the cell viability of (**A**) HN3, a metastatic squamous cell carcinoma of pharynx from lymph node site, (**B**) HepG2 cell, an immortal cell line derived from the liver tissue, (**C**) SKOV3, an ovarian cancer cell line derived from the ascites, and (**D**) TOV21G, ovarian primary malignant adenocarcinoma. MTT assay of four cancer cell lines treated by MC extract at varied concentrations (0-1 mg/mL) for 48 h. Data are representatives of three individual replicates.


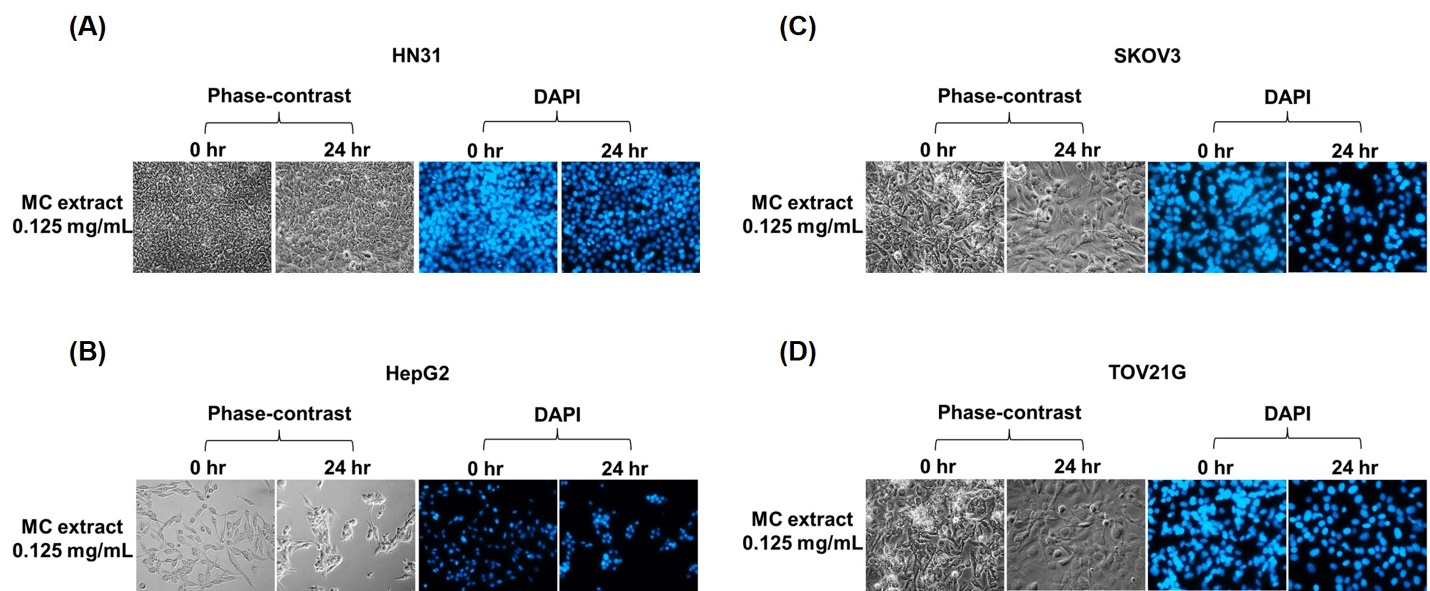


**Supplementary Fig 2.** Effects of the leaf extract of MC on the cell morphological changes and nuclear fragmentation of (**A**) HN31 cells, (**B**) HepG2 cells, (**C**) SKOV3 cells, (**D**) TOV21G. Cancer cells were treated with 125 µg/mL of MC extract for 24 h. Micrographs were taken at 0 and 24 h by a bright-field microscope (phase-contrast) and a fluorescent microscope to observe DNA fragmentation stained by DAPI (blue). Data are representatives of three individual replicates.


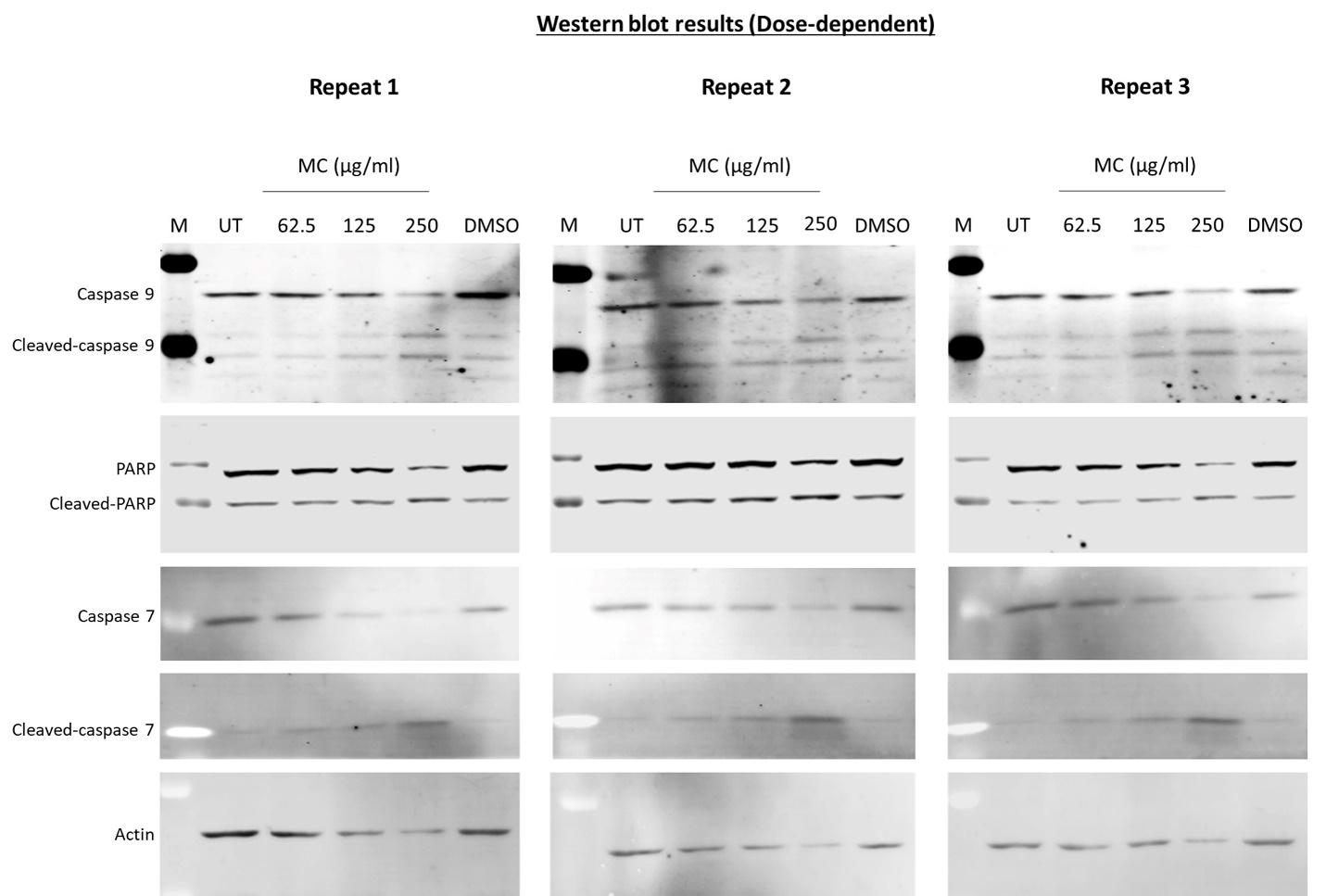


Supplementary Fig 3. Three individual replicates of Western blot analysis of HeLa cells treated with different concentrations of MC extract showing caspase 9, cleaved (activated) caspases 9, caspase 7, cleaved (activated) caspases 7, PARP, cleaved PARP, and actin. DMSO was used as a vehicle control. M = protein markers.

**
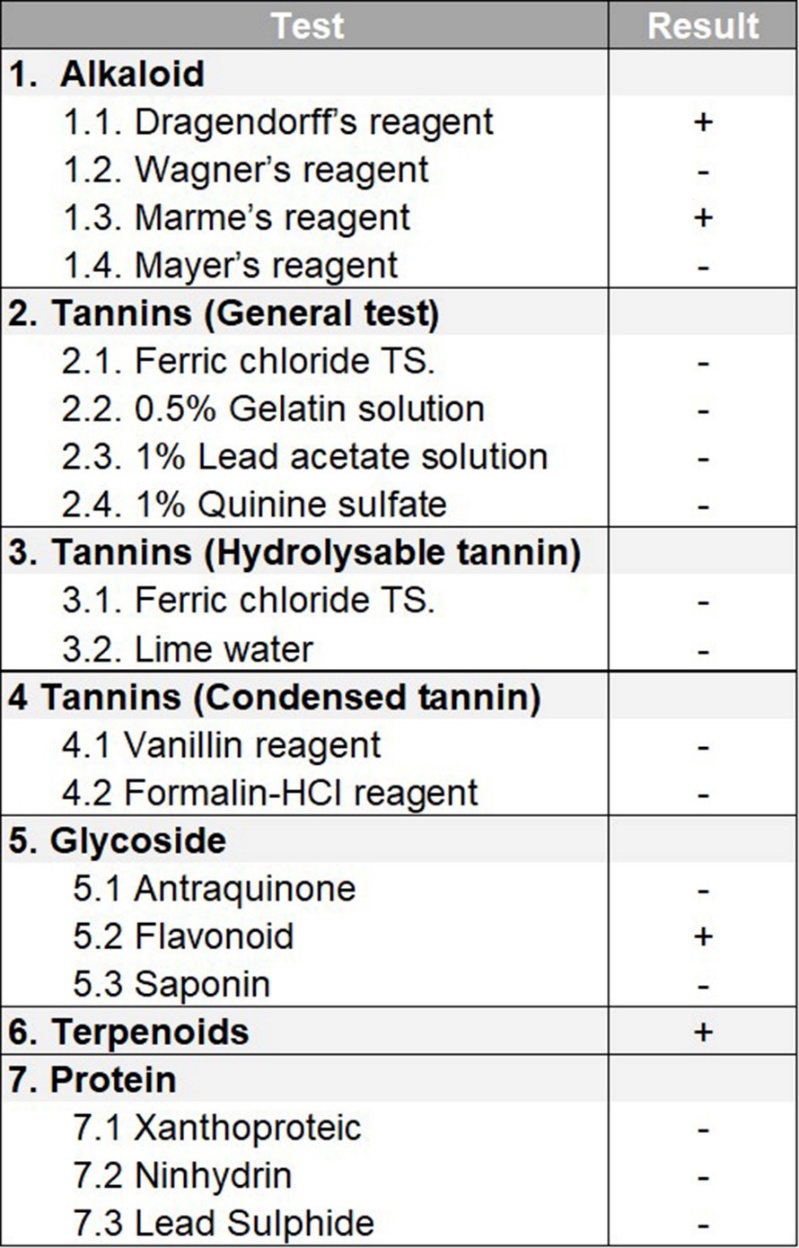
**

**Supplementary Table 1.** Phytochemical tests were evaluated for major chemical groups in MC extract; e.g. flavonoids, alkaloids, glycosides, proteins, terpenoids using chemical reagents. (**+**) Positive result; (**-**) Negative result.
